# The draw of home: How does teacher’s initial job placement relate to teacher mobility in rural China?

**DOI:** 10.1371/journal.pone.0227137

**Published:** 2020-01-30

**Authors:** Yi Wei, Sen Zhou, Yunbo Liu

**Affiliations:** 1 China Institute for Educational Finance Research, Peking University, Beijing, China; 2 Faculty of Education, Beijing Normal University, Beijing, China; Xiamen University, CHINA

## Abstract

Across the world, certain schools struggle to recruit and retain qualified teachers. This study explores teacher mobility across schools in rural China. Using a dataset from the Gansu Survey of Children and Families, this study investigates how teacher’s initial job placement relates to teacher mobility across schools. The findings show that non-local teachers whose initial placements were not in their hometowns were more likely to switch schools. Non-local teachers were also more likely to move for family reasons, compared to moving for personal development or due to involuntary transfer by the local government. The findings suggest that localized recruitment and deployment of teachers can be valuable for reducing teacher mobility rate and retaining teachers in hard-to-staff areas.

## Introduction

A substantial amount of research suggests that, among all school resources, high-quality teachers are crucial determinants of student achievement [1–3]. However, there are large disparities in access to qualified teachers across schools. Abundant evidence demonstrates that students in underdeveloped areas, low-achieving students, and students from marginalized ethnic groups have less qualified teachers than more advantaged students [2,4–6]. Governments in many countries have tried various approaches to recruit high-quality teachers for hard-to-staff schools, including offering financial incentives [7–10], shifting to alternative teacher hiring strategies [7–10], and targeting talented young people for limited terms at hard-to-staff schools (e.g.,“Teach for America” program in U.S.).

However, a number of studies on teacher mobility have shown that efforts focused solely on recruiting teachers are often ineffective, as highly qualified teachers tend to sort themselves from high-poverty, low-performing schools to schools that serve more advantaged students [11–14]. Chudgar and colleagues’ [8] review of research found that although alternative teacher hiring strategies can address teacher shortages in hard-to-staff schools in the short term, they may not improve long-term equity outcomes due to greater teacher attrition, diminished teacher morale, and deprofessionalization of teaching. Studies on programs such as Teach for America (henceforce TFA) found that more than half of TFA teachers exit teaching after two years, and over 80% exit teaching after three years [15].

The uneven distribution of qualified teachers is a major concern in rural China [16–18]. Policies addressing the shortage of qualified teachers in remote rural areas have been issued, including the Free Teacher Education (FTE) policy, the Special Post Teacher (SPT) policy, and a teacher rotation policy. The FTE uses free college education and guaranteed job opportunities to attract high-achieving high school graduates to enroll in teacher education programs during college. Students are required to serve in rural schools for two years after graduation. The SPT uses alternative recruitment strategies to attract college graduates to become teachers in impoverished rural schools. It does not require college graduates to be trained in traditional teacher education programs, and all graduates who pass teacher certification test are eligible for application. The teacher rotation policy encourages or requires public school teachers and principals from high-performing schools to transfer to hard-to-staff or low-performing schools for a certain period of time ranging from months to years during their careers. In 2018, the SPT was the main source of novice teachers in a number of China’s under-developed provinces. Studies have found that although many of the teachers recruited through the SPT did teach in remote rural schools, a significant number left teaching or transferred to other schools close to county seats when they become regular teachers upon completion of a three-year contract [19,20]. Recent studies have found similar results regarding the FTE policy [21,22]. As a result, rural schools experience even higher teacher turnover, and students in these schools are taught by less experienced and qualified teachers.

Why do efforts to recruit and allocate teachers to hard-to-staff schools often failed? Despite first job placements of teachers that match less-qualified teachers with hard-to-staff schools and underdeveloped areas, the lack of high-quality teachers in these schools and areas could result from teacher turnover, including teachers who exit teaching and transfer to other schools. Most prior studies on the sorting of teachers have focused on the job search process and initial matching of teachers to jobs. This study approaches the problem differently by focusing on teacher mobility across schools after the initial match. In this study, using a dataset from the Gansu Survey of Children and Families (GSCF) as a case, we explored the relationship between teachers’ initial job placements and teacher mobility in rural China. The findings indicate that, in addition to recruiting new teachers for hard-to-staff schools, it is equally crucial to retain teachers in rural areas via localized recruitment and deployment of teachers.

## Literature review

### Factors explaining teacher mobility

Research consistently shows that teachers respond to wages, and that higher wages reduce the likelihood of teacher turnover [23–25]. In the meantime, nonpecuniary factors also affect teacher distribution across schools. Research finds that teachers prefer to work in schools and districts with better working conditions, supportive networks of colleagues, lower enrollment and smaller class size, and high-achieving students [11,26,27]. These factors are closely related to teachers’ decisions about where they live and work.

Two aspects of teachers’ preferences about where to live and work help to explain the sorting of teachers across schools and areas. On one hand, teachers prefer to live and work in developed areas with higher wages, better amenities and more educational opportunities for their children. Luschei and Chudgar [28] found that in some cases, the lure of the city trumped teachers’ desire to work close to their homes and families. As a result, schools in underdeveloped areas with challenging living and working conditions can find it difficult to attract and retain qualified teachers.

On the other hand, teachers prefer to teach close to their homes and families [29–32]. Some teachers value working with students who come from a similar background as themselves over students with higher achievement or more advantaged socioeconomic status. Some find feelings of familiarity and closeness to the communities where they grew up to be more important than being in a wealthy environment [29–32]. In examining teacher mobility in India, Mexico and Tanzania, Luschei and Chudgar [28] found that the desire to be close to home was the most common reason teachers sought transfers to new locations or left schools before they completed their teaching commitments. The draw of home is likely to put some students in under-developed areas at a disadvantage, because these areas are less likely to produce college graduates, including prospective qualified teachers.

Sometimes, the draw of home also has its positive side. Studies examining teacher mobility in rural China have found that teachers who were local residents were more likely to have stronger community ties [33], more likely to stay despite tough living and working conditions [34,35], and more likely to hold higher expectations for students [34]. As a result, rural students with local teachers were more likely to have higher aspirations and better achievement [36,37]. These findings suggest the importance of recruiting teachers of local origins for retaining teachers and improving student learning.

In addition to teachers’ preferences about where to live and work, national and regional policies on recruiting, hiring, and allocating teachers also affect teachers’ flexibility in choosing where to teach. In the United States, teacher labor markets are geographically small and localized, thus teacher recruitment decisions are decentralized and largely made by local school leaders [38]. In Japan and Korea, teachers are civil servants hired at the country or regional level. The central or regional governments assign teachers to schools that are in need of teachers and rotate teachers among schools periodically to guarantee the equal distribution of teachers within the region [39,40]. In many European countries, where teachers are also civil servants, governments can assign teachers to particular schools or compel teachers to move between schools if necessary [41]. China represents a case that falls between highly centralized systems, such as South Korea, and decentralized systems, such as the United States.

### Teacher mobility in the Chinese context

In rural China, the county governments assume the main responsibility of financing primary and secondary public schools. The school districts, which correspond to the area of the township, is the basic unit of the educational management in rural areas. In the following paragraphs, we refer to the unit of township educational management as school district. The school district superintendent is in charge of managing the local primary and secondary schools. The authority of hiring, assigning and transferring teachers is shared by different levels of governments, including district superintendents, township governments and county governments. The county governments hire public primary and secondary school teachers according to the demands reported by township governments and assigned teachers to school districts. Recruitment is based on candidate lists or competitive examinations which vary across counties. Usually, only a certain number of successful candidates are selected for a limited number of teaching positions in public schools. Sometimes the selected candidates can express their preference regarding the locations and schools they wish to work, but the final decision is taken by the county and township governments. Usually, the county education bureau would take into consideration the location of teachers’ hometowns when assigning them to school districts. After teachers are assigned to districts, the township governments make the decision to assign them to schools. Sometimes the district superintendents can have some influence on the assignment. Schools do not have the autonomy to hire standard teachers directly from the labor market, because standard teachers in public schools are public employees who are on the government payroll and take officially budgeted posts (bianzhi), which is similar to teachers with civil servant status in some countries. Most teachers recruited by local governments are residents of the counties to which they are assigned and graduated from county-level secondary teachers schools (zhongdengshifan) or regional-level teachers colleges. In addition to standard teachers with *bianzhi*, schools or villages can hire contract teachers, and are responsible for paying them. The process of hiring contract teachers is similar to open recruitment. The vacant positions are filled by candidates applying for employment on a school-by-school basis.

With regard to teacher transfer between schools, authorization is shared between the county education bureau, the superintendent, and the township government. The county education bureau controls teacher transfer to schools located in the county seat and between townships. The county government seat is one of the townships within a county in a rural area, which is often the economic and cultural center of the county. As a result, schools located in the county government seat are better. In some counties, teacher transfer to schools in the county government seat operates through examinations specifically designed to recruit teachers from schools in other towns within the county. If teachers want to transfer to other schools within the same town, they need to get permission from the school district superintendent and township government, and sometimes the county education bureau. Studies in rural areas show that teacher transfer is sometimes used by the local governments as a way of rewarding and punishing teachers. Poor-performing teachers are assigned to schools in remote rural areas, while senior teachers and high-performing teachers have more chances to teach at central schools, schools near county seats, and schools in urban areas [34,35,42,43]. Frequent use of teacher transfer as a means of reward and punishment tends to exacerbate gaps in teacher quality between schools, harming schools that are already disadvantaged in other ways.

In this study, using rural China as a case, we are mainly interested in how teacher’s initial job placement relates to teacher mobility across schools. According to the prior case studies in rural China, teachers who are local residents are more likely to stay, whereas non-local teachers are more likely to move. Thus, we examine the draw of home assumption by comparing teachers whose first job placements were outside of their hometowns and teachers whose first job placements were in their hometowns. We ask:

1. How does the first job placement relate to the likelihood and the timing of the teacher switching schools?
2. How does the first job placement relate to teachers’ reasons for switching schools?

## Data and methods

### Data sources

We used data from the Gansu Survey of Children and Families (GSCF), a longitudinal, multilevel survey of rural children in Gansu province. Gansu is located in interior northwestern China. The per capita GDP of Gansu was ranked 30 out of 31 provinces in 2017. About 76% of the population lives in rural areas. Most children attend primary school in their village, and secondary schools in their town. In the year 2000, the GSCF sampled 2,000 children aged 9 to 12 from 100 villages in 42 districts in 20 randomly sampled counties. To examine rural children’s schooling, achievement, and welfare, the GSCF also included linked surveys with parents, homeroom teachers, school principals, and village leaders from the villages of sampled children. In addition, a teacher questionnaire was administered to all teachers in schools attended by sampled children, providing detailed information on the background of teachers, their job status, remuneration, and time allocation. The second and third wave of the GSCF tracked the same children in 2004 and 2007. To explore how teachers’ preferences for school location affect teacher mobility, we drew on the teacher survey from the 2007 wave of the GSCF. 2,382 teachers participated in the survey, and one teacher did not report his mobility history. Among the 2381 teachers who had mobility status, 2,369 teachers from 198 schools in 44 towns across 20 counties had no missing data on individual variables. Among these teachers, 26 teachers did not have district information. Thus the final sample was 2343.

### Data measures

#### Outcome measures

In the teacher survey, teachers were asked about when they started teaching, when they left and when they moved to another school. Table 1 describes the status of teacher mobility based on these responses. On average, about 55.4% of teachers participating in the 2007 survey had switched schools at least once, and 23.6% of the teachers had switched schools more than twice. 53.7% of those who switched schools did so within the first 5 years, 28.3% after 10 years, 15.6% after 15 years, and 8.2% after 20 years of starting work at their first schools.

**Table 1:** Description of teacher mobility status.

	Total	Percentage
**Total sample**	2381	100%
**Switched at least once**	1318	55.4%
**Ever switching schools**		
0	1063	44.7%
1	756	31.8%
≥ 2	562	23.6%
**Years until the first move**		
≤ 5	708	53.7%
≥ 10	373	28.3%
≥ 15	205	15.6%
≥ 20	107	8.2%

First, we generated two outcome variables measuring teachers’ mobility status. The first is a binary variable, which equals 1 if a teacher had switched schools at least once and 0 if not. The second is a categorical variable, which equals 1 if a teacher had not switched schools, 2 if a teacher had switched schools once, and 3 if a teacher had switched schools more than once.

Second, using a subsample of teachers who had switched schools, we generated two variables measuring the timing of teachers switching schools. One is a binary variable indicating an early career move, which equals 1 if a teacher switched schools for the first time within the first 5 years, and 0 if a teacher switched schools after 5 years. The other is a continuous variable indicating years a teacher stayed at the first school until he/she moved to another school.

In addition, the 2007 survey asked teachers why they left a school. The multiple response options included personal reasons (for example, in order to live with one’s family), better working conditions, better living conditions, and transfer by the local government. The answers indicate whether a specific teacher’s mobility was voluntary or involuntary. Table 2 describes the reasons for moving to other schools based on these responses. About 25% of the 1268 teachers who had switched schools at least once indicated that they moved for family reasons. About 18% of teachers indicated that they moved for better working or living conditions. Over time, the percentage of teachers who moved for family reasons decreased, while the percentage of teachers who moved for personal development stayed the same. On the other hand, about half of the teachers indicated that they were assigned to other schools by local government at first. Over time, the percentage of teachers who were transferred by local government increased from 48% to 70%. The descriptive data indicate that involuntary transfer by the education bureau was the most common reason for teacher mobility in the GSCF survey. Among the other reasons, moving for family was more common than moving for better working conditions or better living conditions.

**Table 2:** Reasons for moving to other schools in the 1st, 2nd, 3rd, and 4th moves (%).

Reason	1st move	2nd move	3rd move	4th move
**Family reasons**	24.53	15.91	9.91	10.61
**Better working condition**	14.12	13.36	6.9	15.15
**Better living condition**	3.55	4.32	3.45	1.52
**Transfer by county education bureau**	5.91	4.32	4.31	3.03
**Transfer by district education office**	42.51	53.44	68.53	66.67
**School consolidation**	3.79	3.93	3.02	.
**Other**	5.6	4.72	3.88	3.03
**Total**	1268	509	232	66

Based on the responses, we generated a binary variable, which equals 0 if a teacher indicated that he or she was involuntarily transferred to another school by the government and 1 if a teacher moved voluntarily for family, career development, better working or living conditions. We generated another binary variable, which equals 1 if a teacher moved voluntarily for family reasons and 0 for better working or living conditions.

#### Variable of interest

The survey asked teachers about the location of their first school and their place of birth. According to the career history provided by the answers, we generated a variable, which equals 1 if the teacher’s first job placement was outside of the district where the teacher was born, and 0 otherwise. We used district rather than village as the measure of local origin because the school district corresponds to the area of the township and is the basic unit of educational management in rural areas. During the time of the survey in rural Gansu, teachers were hired by county governments and allocated to townships rather than particular schools. In this study, we refer to teachers whose first job placement was not in their home districts as non-local teachers, while teachers whose job placement was in their home district as local teachers. According to the prior case studies in rural China, teachers who are local residents are more likely to stay, whereas non-local teachers are more likely to move. We identify “the draw of home” by comparing the probability of school transfer between non-local and local teachers. The hypothesis is that non-local teachers tend to have higher mobility rate than local teachers, and they are more likely to move for family reasons (e.g. to live with family).

Table 3 provides descriptive statistics of teacher-level variables, including gender, marital status (1 = single and 0 = otherwise), age, teacher certification (1 = certified and 0 = otherwise), results of certification exam (1 = scored above 80 and 0 = scored lower than 80), education background at entry to teaching (1 = college degree and 0 = lower than college), normal school education (1 = graduated from normal school and 0 = otherwise), teachers’ employment status (1 = contract teacher and 0 = standard teacher) and grade (1 = teaching middle school and 0 = teaching primary school and kindergarten). The descriptive statistics show that 56% of teachers were not local residents in the districts of their first job placement. The average age was 35.7 years old. About 60% of the teachers were male and 16% were single. 49% taught at secondary schools. 37% got 3- or 4-year college degree when they began teaching and 61% graduated from normal schools. 95% got teacher certification and 70% scored above 80 when taking the teacher certification examination. 9% were contract teachers.

**Table 3:** Descriptive statistics of teacher-level variables.

Variable	Mean	Std. Dev.
**First job placement outside home district**	0.56	0.50
**Male**	0.60	0.49
**Age**	35.70	9.70
**Single**	0.16	0.36
**Teach middle school**	0.49	0.50
**College degree**	0.37	0.48
**Normal school**	0.61	0.49
**Teacher certification**	0.95	0.21
**Certification exam score≥80**	0.70	0.46
**Contract teacher**	0.09	0.29

### Data analysis

First, we used binomial logit models to examine how a teacher’s first job placement related to teacher mobility. The two outcome variables measure whether or not, and the frequency a teacher had switched schools. Second, we used binomial logit model and OLS regression to examine how the first job placement related to the timing of teachers leaving a school. The binary outcome variable indicates early career moves and the continuous outcome variable indicates years of a teacher stayed at the first school until moving to another school. Next, we used binomial logit model to examine the relationship between a teacher’s first job placement and the teacher’s reason for leaving a school. The two outcomes variable indicates whether or not a teacher moved voluntarily, and whether or not moved for family reasons. The variable of interest in the above analysis is teacher’s first job placement. Finally, we used binomial logit model to examine whether non-local teachers who indicated he/she moved for family reasons actually moved back to home districts. The analysis sample is non-local teachers who had switched schools at least once. The outcome variable indicates whether teachers worked in home districts during the time of the survey. The variable of interest is moving for family reasons.

The empirical model is as follows:

$$F(Y_{ij})=\alpha +\beta T_{ij}+C_{k}+\epsilon_{ij}$$

*T_ij_* is a set of teacher characteristics (Table 3). *C_k_* is county fixed effects. The 2007 GSCF survey only asked about the working conditions of the current schools, therefore we cannot capture the characteristics of the prior schools. According the GSCF survey data, more than 93% of teachers work at schools in their home county, 51% in their home district, and 23% in their home village. That is, most of the transfers occur between or within districts within the same county. Therefore, we use county fixed effects to eliminate cross-county variation and compare teachers within a county, and cluster standard errors at the district level instead to account for the common factors which might affect teacher transfer within the same county.

## Results

### How does the first job placement related to teacher mobility?

Table 4 shows the relationship between the first job placement and teacher mobility status. All of the models use county fixed effects with standard errors clustered at the district level. The results are presented in odds ratios. Column 1, 2 and 3 uses the full sample to examine whether non-local teachers, those whose first job placements were outside home district, were more likely to switch schools. Column 4 and 5 use subsample of teachers who had switched at least once. Column 4 examines whether non-local teachers tended to move early in their teaching career. Column 5 examines the relationship between first job placement and the years a teacher stayed at the first school until moving to another school.

**Table 4:** Estimation results of the relationship between first job placement and teacher mobility status.

	Whether or not move	Frequency of move		Timing of move	
		Never moved	Moved more than once	Early move	Years until the first move
	1	2	3	4	5
**First job placement outside home district**	2.421***	0.455***	1.376*	1.073	-0.781*
	(0.335)	(0.070)	(0.219)	(0.155)	(0.363)
**Male**	1.290*	0.926	1.761***	1.119	0.298
	(0.163)	(0.129)	(0.216)	(0.202)	(0.312)
**Age**	1.046***	0.968***	1.028***	0.870***	0.430***
	(0.010)	(0.009)	(0.008)	(0.011)	(0.022)
**Single**	0.570**	1.395+	0.275***	4.442**	0.536
	(0.107)	(0.255)	(0.082)	(2.404)	(0.364)
**Teach middle school**	0.551***	1.329	0.414***	0.882	-1.022**
	(0.098)	(0.235)	(0.089)	(0.164)	(0.317)
**College degree**	0.405***	2.095***	0.559***	1.275	-0.318
	(0.055)	(0.289)	(0.095)	(0.327)	(0.302)
**Normal school**	1.044	0.948	0.981	1.095	-0.731*
	(0.128)	(0.122)	(0.150)	(0.123)	(0.341)
**Teacher certification**	1.655+	0.678	1.562	2.391+	0.053
	(0.448)	(0.197)	(0.778)	(1.080)	(0.765)
**Certification exam score≥80**	1.161+	0.876	1.092	0.929	-0.266
	(0.102)	(0.085)	(0.146)	(0.139)	(0.379)
**Contract teacher**	0.824	1.166	0.933	0.809	-0.542
	(0.167)	(0.259)	(0.250)	(0.264)	(0.634)
**N**	2343	2343	2343	1294	1294

Notes: Column 1, 2 and 3 used the full sample; Column 4 and 5 used subsample of teachers who has switched schools at least once; + p<0.10

* p<0.05

** p<0.01

*** p<0.001.

The coefficient of the first job placement in Column 1 shows that non-local teachers were 2.4 times more likely to switch schools compared to local teachers. When holding other factors constant, male teachers were 1.3 times more likely to switch schools than female teachers. As for teachers assigned to schools outside their home district, there was no difference in likelihood to move between male and female teachers (the results are not presented in Table 4). Having a college degree when entering teaching, being single, and teaching in middle schools were associated with a lower probability of switching schools. This differs from prior findings that teachers with a higher level of education tend to have higher turnover rates [4,6,23,44]. One possible explanation is that teachers with college degree tend to have more choices when local government make deployment decision. These teachers are also more likely to be assigned to schools with better quality and location. On the other hand, teachers without college degree have less choice and are more likely to be assigned to schools in remote rural areas. As a result, these teachers have stronger motivation to seek mobility. Further analysis of GSCF’s data partially confirms the explanation, finding that teachers without college degree at entry were more likely to switch schools after they acquired college degree through in-service training.

Column 2 and 3 present the results of teachers who stayed at the same school and teachers who had moved multiple times compared to teachers who had moved once. The results show that non-local was 1.4 times more likely to switch schools more than once and 54.5% less likely to stay compared to local teachers. When holding other factors constant, male teachers were more likely to move more than once. Single teachers, teachers with college degree and teachers in middle school were less likely to move more than once.

Column 4 and 5 present the estimation results of the relationship between first job placement and the timing of switching schools. According to the results in column 3, there is no difference in the likelihood of moving early in one’s career for a non-local teacher compared to teachers who were residents. According to the results in column 4, among teachers who have switched at least once, local teachers tended to stay at their first school longer than non-local teachers. Together, the results of column 3 and 4 indicate that although the non-local teachers were not more likely to switch schools within the first 5 years of teaching career, they tended to teach at the first school for a shorter period.

### How does the first job placement relate to why teachers move?

Table 5 shows the relationship between first job placement and teachers’ reasons for switching schools. All of the models use county fixed effects with standard errors clustered at the district level. The results are presented in odds ratios. Columns 1 and 2 use the full sample to examine whether non-local teachers were more likely to apply for transfer rather than transferred by local governments. Column 2 adds an interaction term between male and first job placement to examine whether there was heterogeneity between male and female teachers when their first job placements were not in home districts. The results show that non-local teachers were more likely to apply for transfer themselves. Holding other factors constant, female teachers were more likely to apply for transfer. According to column 2, there is no significant difference in the likelihood of voluntary transfer between male and female teachers when their first job placements were outside home districts.

**Table 5:** Estimation results of the relationship between first job placement and reasons to move.

	Move voluntarily		Move for family reasons	
	1	2	3	4
**First job placement outside home district**	3.269***	2.472***	2.052*	1.251
	(0.500)	(0.459)	(0.677)	(0.435)
**Male**	0.770*	0.553*	0.687+	0.356**
** **	(0.089)	(0.141)	(0.154)	(0.128)
**Male◊First job placement**		1.567		2.297*
** **		(0.454)		(0.917)
**Age**	1.021+	1.023*	1.024	1.028
	(0.011)	(0.011)	(0.018)	(0.018)
**Single**	0.306***	0.311***	0.501	0.537
	(0.069)	(0.070)	(0.236)	(0.252)
**Teach middle school**	0.786+	0.776+	0.799	0.777
	(0.103)	(0.103)	(0.182)	(0.177)
**College degree**	0.676**	0.680**	0.746	0.757
	(0.089)	(0.090)	(0.209)	(0.208)
**Normal school**	0.995	0.991	0.837	0.842
	(0.107)	(0.108)	(0.142)	(0.144)
**Teacher certification**	1.717	1.734	2.599	2.736
	(0.602)	(0.608)	(1.777)	(1.853)
**Certification exam score≥80**	1.067	1.063	0.953	0.96
	(0.132)	(0.132)	(0.143)	(0.151)
**Contract teacher**	1.502	1.474	1.925	1.845
** **	(0.396)	(0.385)	(1.333)	(1.268)
N	2343	2343	531	531

Notes: Column 1 and 2 used the full sample; column 3 and 4 used subsample of teachers who switched the first time; Column 2 and 4 control for the interaction term of male and first job placement; columns 3 and 4 use subsample of teachers who moved voluntarily;+ p<0.10

* p<0.05

** p<0.01

*** p<0.001

Columns 3 and 4 use subsample of teachers who move voluntarily to examine whether non-local teachers were more likely to apply for transfer due to family reasons. Column 4 adds an interaction term between male and first job placement to examine whether there was heterogeneity between male and female non-local teachers. The coefficient of the first job placement shows that a non-local teacher was more likely to move for family reasons compared to moving in pursuit of a better environment or personal development. In general, male teachers were less likely to move voluntarily for family reasons. The coefficient of the interaction term in column 4 indicates that when male teachers were first assigned to schools outside their home districts, they tended to apply for transfer for family reasons.

Because “move for family reasons” does not imply that teachers who reported their reasons to switch schools had moved back to home districts, we use subsample of non-local teachers who had switched schools at least once to examine whether, after the first move, those teachers who indicated that they switched schools in order to live with their families were actually closer to their home districts. The results in Table 6 show that non-local teachers who indicated that they moved to other schools were 1.5 times more likely to work in home district at the time of the survey, which suggests the existence of “draw of home effect”.

**Table 6:** Estimation results of the relationship between “move for family” and current work location.

	Work in home district
**Move for family**	1.539*
	(0.304)
**Male**	1.693
	(0.692)
**Age**	1.063***
	(0.018)
**Single**	1.305
	(0.496)
**Teach middle school**	1.625*
	(0.377)
**College degree**	0.894
	(0.198)
**Normal school**	0.998
	(0.191)
**Teacher certification**	2.717
	(1.913)
**Certification exam score≥80**	0.835
	(0.168)
**Contract teacher**	0.424
	(0.400)
**N**	786

Notes: The analysis used the subsample of non-local teachers who switched schools for the first time, including those teachers who move only once, and the first move of those who had moved multiple times; + p<0.10

* p<0.05

** p<0.01

*** p<0.001.

## Conclusion and discussion

This study aimed to examine how teachers’ first job placement associated with teacher mobility across schools. Regarding the first question “How does the first job placement related to the likelihood and the timing of the teacher switching schools?”, the findings show that non-local teachers whose first job placements were outside of home districts were more likely to switch schools compared to local teachers whose first job placements located within home districts. In terms of the timing, there was no difference in the likelihood of moving early in one’s career for a non-local teacher compared to local teachers. On the other hand, being assigned to a school outside the home district was likely to reduce the probability of a late-career move.

Regarding the second question, “How does the first job placement relate to teachers’ reasons for switching schools?”, the findings show that non-local teachers were more likely to apply for transfer instead of involuntary transfer by government. Non-local teachers also tended to move for family reasons instead of moving for personal development. We also examined whether, after the move, those teachers who indicated that they switched schools in order to live with their families were actually closer to their home districts. The results partially support the existence of “draw of home effect”.

In addition, we also examined the gender difference in the relationship between first job placement and teacher mobility. There are no differences between female and male teachers in the likelihood to transfer schools and the frequency. In terms of reasons for transfer, in general, male teachers were less likely to apply for transfer themselves when their first job placements were not in their home districts. However, among the non-local teachers who apply for transfer, male teachers were more likely to do so for family reasons.

The lack of qualified teachers and teacher attrition in hard-to-staff schools are important issues worldwide. Governments in many countries have tried various approaches to address these problems. Our findings have implications for the design and implementation of policies to address teacher shortages and improve teacher quality in hard-to-staff schools. Our study finds that, compared to local teachers, non-local teachers tended to have higher mobility rate. They were more likely to apply for transfer largely because they wanted to move back to live with their families. The findings suggest that localized recruitment and deployment of teachers have value in retaining teachers and reducing teacher turnover rate. Our findings also suggest the importance of localized teacher preparation systems in underdeveloped rural areas, as these areas produce a lower proportion of college graduates, and thus fewer potential qualified teachers, than do urban and developed areas. Recent policy reforms carried out by the government, such as SPT and FTE programs, and efforts initiated by NGOs such as Teach for China (modeled after Teach for America), all focus on recruiting new college graduates as alternative ways of staffing rural schools and raising teacher quality. However, these efforts fall short of addressing the problem of teacher retention in rural schools. Despite the increasing emphasis on teachers’ educational level, policies should reconsider the role of county-level secondary teachers schools and 3-year teachers colleges in admitting local students and training local teachers.

The findings of this study are limited in several ways. First, the primary data for this study were drawn from the GSCF survey conducted in rural Gansu, thus the findings mainly reflect rural schools in counties characterized by poverty, like Gansu. Second, because of the way teachers were sampled in the GSCF survey, we do not have information about those teachers who left teaching. The 2007 GSCF survey asked about the number of teachers left schools and the status of those teachers after they left the school, including retired, taught in other schools, exited teaching. In 2006/2007 academic year, the average percentage of teachers leaving a school was 6.0% (343 teachers in total). Among those teachers leaving, 5.8% of teachers exit teaching. Therefore, attrition due to teachers exit teaching would not cause significant bias. Regarding the limitations of the current study, future studies on teacher mobility may consider using administrative data that are linked to schools and cover all teacher records within a county to account for teachers who left teaching. In addition, for a country as vast and diverse as China, more studies are needed to understand the differences and similarities in patterns of teacher mobility across regions.

## Supporting information

S1 Appendix School-level independent variables from 2000, 2004, and 2007 GSCF survey. (DOCX)
