# Supplementary material for: The draw of home: How does teacher’s initial job placement relate to teacher mobility in rural China?
Source: PLoS One. 2020 Jan 30;15(1):e0227137. doi: 10.1371/journal.pone.0227137 (PMC6992212; doi:10.1371/journal.pone.0227137)
Supplement: S1 Appendix — (DOCX) [file pone.0227137.s001.docx]

**Appendix** School-level independent variables from 2000, 2004, and 2007 GSCF survey

| Variable | Type | Description |
| --- | --- | --- |
| **School-level variables from principal survey** | | |
| Central school | Dichotomous | 1= central school and 0= otherwise |
| Boarding school | Dichotomous | 1= boarding school and 0= otherwise |
| Primary school | Dichotomous | 1= primary school and 0= otherwise |
| Number of classrooms | Continuous |  |
| % classrooms with rainproof roofs | Continuous |  |
| % dilapidated classrooms | Continuous |  |
| Student enrollment | Continuous |  |
| % minority students | Continuous |  |
| % minority teachers | Continuous |  |
| % regular teachers | Continuous |  |
| % teachers with college degree | Continuous |  |
| % teaching experience $\leq$ 5 years | Continuous |  |
| % teaching experience $\geq$ 20 years | Continuous |  |
| Monthly wage of regular teachers | Continuous |  |
| **Teacher variables averaged at school level from teacher survey** | | |
| Mean age of teachers | Continuous |  |
| % teachers who are villagers | Continuous |  |
| % teachers with teacher certification | Continuous |  |
| % teachers with level 1 or senior rank | Continuous |  |
| Average working hours in class per week | Continuous |  |
| Average working hours after class per week | Continuous |  |
